# The biological characteristics of tertiary lymphoid structures in head and neck cancer

**DOI:** 10.3389/fimmu.2026.1818962

**Published:** 2026-05-12

**Authors:** Suwen Bai, Yuan Wei, Fankai Liu, Hexing Sun, Xianhai Zeng, Peng Zhang

**Affiliations:** 1 Department of Otolaryngology, Shenzhen Longgang Otolaryngology Hospital and Shenzhen Institute of Otolaryngology, Shenzhen, Guangdong, China; 2 The Second Affiliated Hospital, School of Medicine, The Chinese University of Hong Kong, Shenzhen & Longgang District People’s Hospital of Shenzhen, Shenzhen, China; 3 The First School of Clinical Medicine, Faculty of Medicine, Yangzhou University, Yangzhou, China; 4 Department of Biomedical Sciences, Division of Biomedical Health Sciences, School of Medicine, The Chinese University of Hong Kong, Shenzhen, Guangdong, China

**Keywords:** biological characteristics, head and neck cancer, health management, immunotherapy, tertiary lymphoid structures

## Abstract

Head and neck cancer (HNC) is one of the most prevalent malignancies worldwide, and its clinical management remains fraught with formidable challenges. In recent years, as our understanding of the tumor microenvironment (TME) has deepened, immunotherapy—especially the clinical application of immune checkpoint inhibitors (ICIs)—has brought a revolutionary breakthrough to HNC treatment. However, only a subset of patients can derive clinical benefits from such therapies, highlighting the urgent need to identify reliable predictive biomarkers. Tertiary lymphoid structures (TLS), lymphocyte aggregates ectopically formed in non-lymphoid tissues such as chronically inflamed or tumor sites with functions analogous to secondary lymphoid organs, have emerged as a burgeoning research hotspot in tumor immunology. This review aims to systematically elaborate on the biological characteristics of TLS in HNC, their clinical value as biomarkers for prognostic evaluation and immunotherapy response prediction, current TLS detection and assessment methodologies, as well as potential therapeutic strategies targeting TLS. We employed a systematic literature review methodology. Studies have confirmed that mature, intra-tumoral TLS are significantly correlated with improved patient prognosis and higher immunotherapy response rates, acting as the “central core” of tumor immunity by initiating and sustaining adaptive anti-tumor immune responses locally. Despite the promising clinical translation prospects of TLS, standardization of assessment systems, development of non-invasive detection technologies, and clarification of TLS functional heterogeneity across different HNC subtypes remain major challenges in current research. This review synthesizes the latest advances in this field, providing a comprehensive and insightful perspective for understanding the pivotal role of TLS in HNC treatment.

## 1 Introduction

Head and neck cancer (HNC) is a heterogeneous group of malignant tumors originating from the mucosal epithelium of the head and neck, primarily including oral cancer, oropharyngeal cancer, hypopharyngeal cancer, laryngeal cancer, and nasopharyngeal cancer, among which more than 90% are squamous cell carcinomas (Head and Neck Squamous Cell Carcinoma, HNSCC) (1). Despite significant progress in traditional treatment modalities such as surgery, radiotherapy, and chemotherapy, the five-year survival rate for patients with locally advanced or recurrent/metastatic head and neck cancer remains suboptimal. Moreover, conventional treatments often come with severe toxic side effects, severely impacting patients’ quality of life (2, 3).

Tertiary lymphoid structures (TLS), also known as ectopic lymphoid structures, are aggregates of lymphocytes induced in non-lymphoid tissues such as chronic inflammation, autoimmune diseases, organ transplant rejection, or tumors, in addition to the formation of lymphoid organs during embryonic development (4, 5). The structure and function of the Tumor-Lymphoid System (TLS) are highly analogous to those of secondary lymphoid organs (such as lymph nodes and the spleen), typically comprising well-defined T-cell and B-cell zones, with the latter potentially developing into B-cell follicles containing germinal centers (GCs). Additionally, the TLS includes mature dendritic cells (DCs), follicular dendritic cell (FDC) networks, and high endothelial microvessels (HEVs), which serve as critical pathways for lymphocytes to migrate from the bloodstream into the TLS (4, 6). This highly organized architecture enables the TLS to perform comprehensive immune functions at the tumor-*in-situ* level: capturing and presenting tumor antigens, activating initial T cells, promoting affinity maturation and class switching of B cells, thereby generating a robust adaptive immune effector cells targeting tumor-specific antigens (7).

In recent years, immune checkpoint inhibitor (ICI), represented by PD-1/PD-L1 inhibitors, have transformed the treatment landscape for advanced head and neck squamous cell carcinoma (HNSCC), delivering durable clinical benefits to some patients (1, 8). However, the overall objective response rate to ICIs remains only 15-20%, indicating the presence of complex immunosuppressive mechanisms within the tumor microenvironment (TME) that limit the broad efficacy of immunotherapy (9). TME can be broadly classified into “immunoinflammatory” (“hot” tumors), “immunorejective, “ and “immune desert” (“cold” tumors) (10). “Hot” tumors are typically characterized by effector T cell infiltration and exhibit better responses to ICI therapy. Transforming “cold” tumors into “hot” tumors represents one of the core strategies for enhancing the efficacy of immunotherapy. Among the various components of the TME, a highly organized aggregate of immune cells-TLS-has been identified as playing a pivotal role.

In this review, we focus on the tertiary lymphoid structures in the treatment of HNC including its formation mechanisms, structural functions, and heterogeneity; secondly, it focuses on analyzing the clinical value of TLS in predicting prognosis and immunotherapy efficacy for head and neck cancer; thirdly, it provides a detailed overview of mainstream and cutting-edge technologies currently used for TLS detection and assessment; finally, it explores innovative therapeutic strategies that may target TLS in the future.

## 2 Biological characteristics of the TLS

### 2.1 Formation and maturation mechanism of TLS

**Figure 1:**
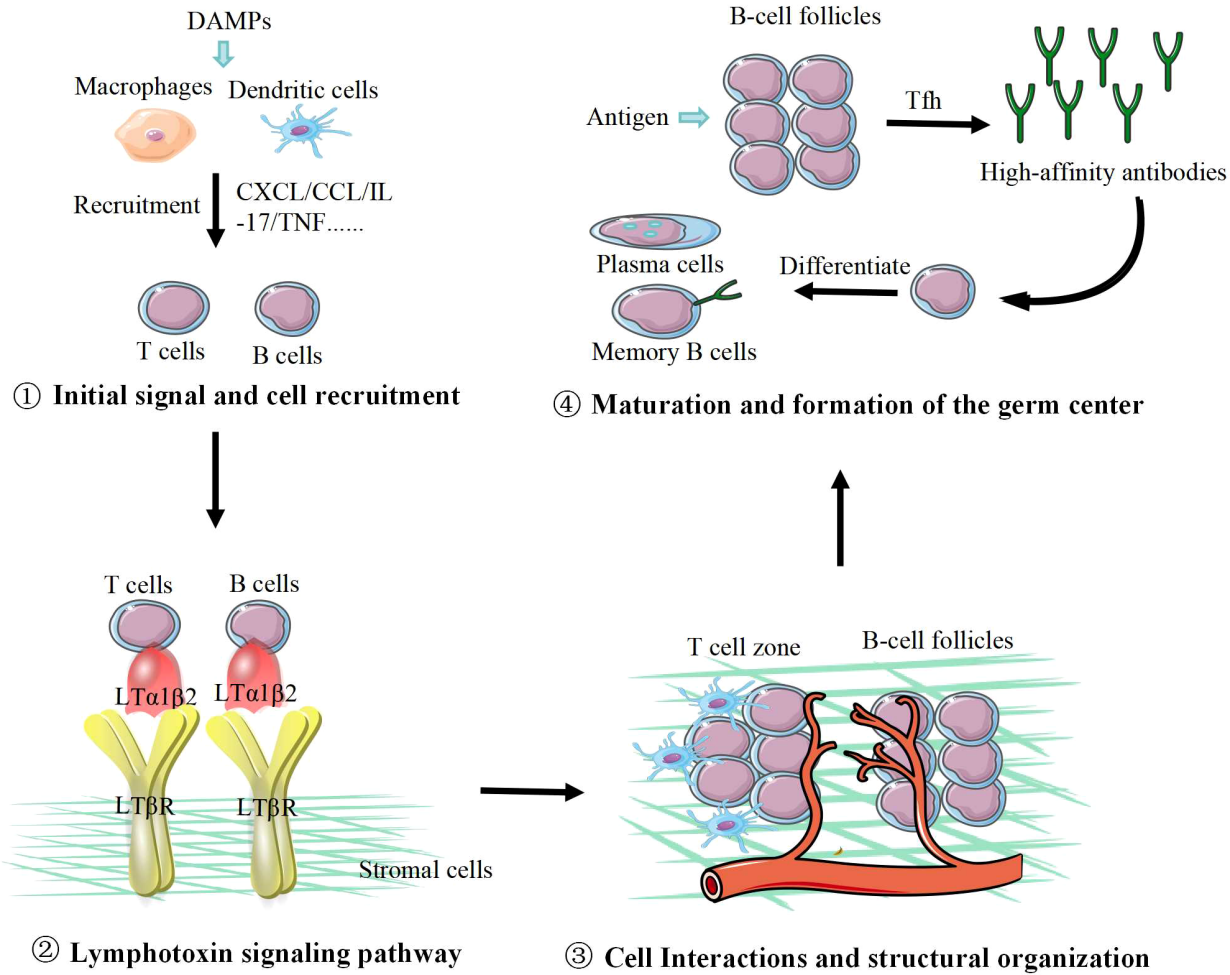
The formation process of tertiary lymphoid structures. ① Tumor antigens collaborate with damage-associated molecular patterns (DAMPs) to activate local innate immune cells, prompting them to secrete pro-inflammatory cytokines and chemokines that recruit B and T cells; ② B and T cells express LTα1β2 on their surfaces, which binds to LTβR on the surface of stromal cells, enabling their colonization within tissue sites; ③ Immune and stromal cells aggregate and interact, gradually forming tertiary lymphoid structures. Concurrently, lymphocytes efficiently migrate through high-endothelial venules, continuously replenishing the TLS lymphocyte pool; ④ Under sustained antigen stimulation, follicular helper T cells (Tfh) facilitate high-affinity antibody production, driving B cells to differentiate into plasma cells and memory B cells, thereby establishing germinal center (GC) formation.

The formation and maturation of TLS is a complex process involving multiple steps, cells, and molecules, with its core mechanism being the reactivation of molecular signaling pathways that mimic lymphoid organ development within the tumor microenvironment (11). As shown in **Figure 1**.

#### 2.1.1 Initial signal and cell recruitment

The death of tumor cells (such as immunogenic cell death) releases tumor antigens and damage-associated molecular patterns (DAMPs), activating local innate immune cells like dendritic cells and macrophages (12). These activated cells, along with tumor and stromal cells, begin secreting a series of pro-inflammatory cytokines and chemokines (13). Among these, the chemokine CXCL13 is considered a key molecule initiating TLS formation (14, 15). It is primarily produced by follicular helper T cells (Tfh) and stromal cells, responsible for recruiting CXCR5^+^ B cells and some T cells (16, 17). Concurrently, chemokines CCL19 and CCL21 secreted by stromal cells and dendritic cells recruit naive T cells and mature dendritic cells via their receptor CCR7, laying the foundation for T cell zone formation (18).

#### 2.1.2 Lymphotoxin signaling pathway

The lymphotoxin (LT) signaling pathway plays a central role in the maintenance and maturation of the T lymphoid site (TLS). Lymphotoxin-α1β2 (LTα1β2) is expressed by lymphoid tissue-inducing (LTi) cells, B cells, and activated T cells, binding to the lymphotoxin-β receptor (LTβR) on the surface of stromal cells. This interaction induces stromal cells to differentiate into lymphoid tissue stromal cells and upregulates multiple molecules critical for lymphocyte homing, survival, and function. These include chemokines such as CCL19, CCL21, CXCL12, and CXCL13, as well as cell adhesion molecules like ICAM-1, VCAM-1, and MAdCAM-1.

#### 2.1.3 Cell interactions and structural organization

As various immune cells and stromal cells aggregate and interact under specific chemokine gradients, the structure of TLS gradually becomes defined (19). T cells and antigen-presenting DCs aggregate to form the T cell zone, B cells aggregate under the guidance of CXCL13 to form B-cell follicles (20). At HEVs, lymphocytes express L-selectin to bind molecules such as PNAd (Peripheral Node Addressin) on HEV surfaces, enabling efficient migration from the bloodstream to continuously replenish the lymphocyte pool within the TLS (21–23).

#### 2.1.4 Maturation and formation of the germ center

Under sustained antigenic stimulation, functional GCs develop within B-cell follicles (24). With the assistance of Tfh cells, B cells within GCs undergo rapid proliferation, high-frequency somatic hypermutation, and class-switch recombination (25, 26). This process selects high-affinity antibodies and differentiates B cells into plasma cells and memory B cells. Mature TLS containing GCs represent the hallmark of local humoral immune responses and signify the highest level of immunological organization (18, 27, 28).

### 2.2 Structure and function of TLS

Mature tumor lymphoid tissue structurally represents a highly concentrated version of secondary lymphoid organs, serving multifaceted functions: It serves not only as a gathering place for immune cells but also as a fully functional “immune reactor” (29). This structure independently executes a series of complex immune processes-including antigen presentation, lymphocyte activation, proliferation, and differentiation-thereby generating and sustaining a diverse, tumor-specific immune effector force locally within the tumor (30). Its core function lies in initiating precise and highly efficient antitumor immune responses at the tumor site (29, 31). The TLS composition and related functions are shown in **Table 1**.

**Table 1:** TLS structure and functions.

Structure	Function
B-cell zone/follicle	This region is crucial for B cell proliferation, differentiation, and antibody affinity maturation, ultimately yielding plasma cells and memory B cells capable of secreting high-affinity antibodies.
T-cell zone	CD4^+^ and CD8^+^ T cells gather, interact with antigen-presenting cells such as dendritic cells, initiate cellular immune responses, and assist B cells in producing antibodies.
Matrix and Vascular Network	Located within B-cell follicles, these cells are not true antigen-presenting cells but can persistently display antigens on their surfaces for B-cell recognition, playing a crucial role in the formation and maintenance of germinal centers. They also form specialized blood vessels serving as the primary pathway for lymphocytes to directly enter TLS tissues, supporting rapid immune cell recruitment.

#### 2.2.1 B-cell zone/follicle

The core region is primarily formed by clusters of CD20^+^ B cells, and it may contain germinal centers. Within germinal centers reside B cells undergoing high-frequency somatic hypermutation and antibody class switching, alongside follicular dendritic cells responsible for capturing and presenting antigens to B cells (32, 33).

#### 2.2.2 T-cell zone

This zone is rich in CD3^+^ T cells, particularly CD4^+^ helper T cells and CD8^+^ cytotoxic T cells, as well as antigen-presenting mature dendritic cells expressing CD83^+^ or LAMP3^+^ (34–37). This serves as the primary site for T cell activation and proliferation. Dendritic cells present antigens captured from tumor cells to naive T cells, initiating the cellular immune response (38).

#### 2.2.3 Matrix and vascular network

This primarily includes the stromal cell network and high endothelial venules. The stromal cell network comprises fibroblast-reticular cells (FRCs) and follicular dendritic cells (FDCs), which form the scaffolding of the TLS and secrete key chemokines and survival factors to guide the localization and interactions of immune cells (39). HEVs are uniquely structured blood vessels expressing specific molecules such as PNAd, serving as the primary gateway for circulating lymphocytes to enter TLS. The density and functional state of HEVs directly influence the TLS’s capacity for immune cell recruitment (32, 40).

### 2.3 The heterogeneity of TLS

TLS is not a homogeneous structure; it exhibits significant heterogeneity across different patients, tumor types, and even distinct regions within the same tumor. This heterogeneity manifests primarily in two dimensions: maturation status and spatial distribution, profoundly influencing its clinical significance.

#### 2.3.1 Maturity heterogeneity

The formation of TLS is a dynamic process with a continuous spectrum of maturity, ranging from simple lymphocyte aggregates to highly organized structures (41). It is broadly categorized into early TLS, primary follicular-like TLS, and secondary follicular-like TLS. Early TLS consists solely of diffuse aggregates of T cells and B cells; primary follicular-like TLS forms distinct T cell and B cell zones without germinal centers; secondary follicular-like TLS contains active germinal centers (GCs), signifying a mature and functionally active humoral immune response is underway, representing the most mature form of TLS (42). Research generally indicates that TLS maturity correlates positively with its functional and prognostic value (43). Mature TLS containing GCs is associated with stronger antitumor immunity, improved patient survival, and higher immunotherapy response rates (44).

#### 2.3.2 Spatial distribution heterogeneity

The spatial distribution of tumor-associated lymphocytes (TLS) in the tumor microenvironment carries distinct biological significance and prognostic value (42). Intra-tumor TLS, which are directly embedded within tumor cell nests or adjacent to tumor cells, are considered to possess the strongest antitumor potential-because their effector cells and antibodies can act on target cells most directly and rapidly (44, 45). Peritumoral TLS are distributed in the stromal regions at tumor margins or invasion frontiers. Although they also exhibit antitumor functions, their effects require longer physical distances or penetration of stromal barriers to reach tumor cells (19, 43). Stromal TLS are located in fibrotic stromal regions distant from the tumor body, and their functions and prognostic significance remain incompletely elucidated, though they are hypothesized to be associated with the chronic inflammatory background of tumors (46, 47).

## 3 The value of TLS in the diagnosis, treatment, and prognosis of HNC

TLS, as a specialized immune structure with quasi-secondary lymphoid organ functions within the tumor microenvironment, demonstrates significant clinical value in the diagnosis, treatment, and prognostic assessment of head and neck cancer. At the diagnostic level, the presence, density, maturity, and spatial distribution characteristics of TLS can serve as potential biomarkers (29). Through methods such as pathological section staining and imaging detection, TLS aids in the early screening, pathological classification, and risk stratification of invasive metastasis for HNC (48). It is particularly valuable for identifying and differentiating occult lesions (49). Therapeutically, TLS can reshape the tumor-suppressive microenvironment by recruiting and activating immune cells such as T cells and B cells (50). Its presence and functional status not only predict patient response to treatments like immune checkpoint inhibitors and chemotherapy but also provide a theoretical basis for novel therapeutic strategies targeting TLS formation to enhance antitumor immune efficacy (51). In prognostic assessment, multiple clinical studies confirm that the presence of TLS in head and neck cancer tissues particularly mature, functional TLS is strongly associated with longer disease-free survival and overall survival (52, 53). This serves as an independent prognostic factor distinct from traditional indicators like tumor stage and differentiation grade, providing crucial guidance for personalized treatment decisions and follow-up management. Next, we will discuss the characteristics and significance of TLS for various HNC including oral cancer, nasopharyngeal carcinoma, laryngeal cancer, and thyroid cancer.

### 3.1 Characteristics and significance of TLS in oral cancer

Oral cancer is one of the common malignant tumors among HNC (54). Research indicates that TLS significantly impacts both pathological outcomes and clinical prognosis in oral cancer patients (55). Peng et al. suggested that TCF1/TCF7^+^ T cell subpopulations in TLS may serve as novel therapeutic targets for modulating immune responses in oral squamous cell carcinoma and hold promise as new prognostic markers (55). Li et al. confirmed the presence of TLS in oral cancer tissues, which are closely associated with the infiltration levels of CD8^+^ T cells and CD57^+^ NK cells-indicating that TLS positivity serves as a significant indicator for favorable prognosis in patients with oral cancer (56). Almangush et al. reported that TLS can predict survival rates for early-stage buccal squamous cell carcinoma and demonstrates independent prognostic predictive capability superior to conventional WHO grading and TNM staging systems (57). Therefore, inducing or enhancing the formation of TLS is considered a potential strategy that holds promise for improving the efficacy of immunotherapies such as immune checkpoint inhibitors in the diagnosis and treatment of oral cancer, thereby opening new therapeutic avenues for enhancing patient prognosis (58).

### 3.2 Characteristics and significance of TLS in nasopharyngeal carcinoma

Similar to other regions of the head and neck, the nasopharynx can develop various types of tumors, including epithelial, mesenchymal, lymphoid, and neuroectodermal tumors (59). However, the most notable among these is nasopharyngeal carcinoma (NPC) - a “special” tumor that differs significantly from other head and neck tumors in many aspects (60). In the diagnosis of NPC, early symptoms are often atypical and easily overlooked. Most patients are diagnosed at an advanced stage, significantly compromising treatment outcomes (61, 62). In terms of treatment, the unique anatomical location of the nasopharynx imposes significant limitations on traditional surgical resection. Furthermore, issues such as radiotherapy-induced side effects and chemotherapeutic drug resistance further complicate clinical management (63). In terms of prognosis, patients with advanced-stage NPC are highly susceptible to metastasis (64). Once metastasis occurs, the prognosis for NPC patients is typically poor. Therefore, appropriate assessment is crucial in the clinical management of NPC. Among these, TLS, a tissue structure formed within a tumor that is structurally and functionally similar to a lymph node, playing a distinct role in diagnosis, treatment, and prognosis. Liu et al., showed that TLS-related cell signatures correlate with prognosis and PD-1 blockade response, offering insights for therapeutic strategies in NPC (65). Hou et al., suggested that TLS-related RNA interacts with metastasis risk factor in NPC (66). Liu et al., reported that TLS formation predict immunotherapy response in NPC (67). In summary, TLS serve as crucial anatomical landmarks for assessing the extent of metastasis and achieving accurate staging in nasopharyngeal carcinoma diagnosis. During treatment, they represent a key region that must be considered when delineating radiotherapy target volumes. Regarding prognosis, their involvement is frequently associated with higher recurrence risk and poorer survival rates. Therefore, meticulous evaluation of level III lymph nodes holds clear clinical significance throughout the comprehensive management of nasopharyngeal carcinoma.

### 3.3 Characteristics and significance of TLS in laryngeal cancer

Laryngeal cancer is a significant malignancy in the head and neck region, with its pathogenesis closely associated with lifestyle factors such as smoking and alcohol consumption (68). Current treatment strategies emphasize precise staging and the development of individualized approaches within a multidisciplinary collaborative framework, where early diagnosis and function-preserving therapy are critical for improving patient outcomes (69). The TLS serves not only as a key prognostic marker for laryngeal cancer but also as a predictive indicator of the efficacy of radiotherapy, chemotherapy, and immunotherapy. For instance, Gkegka et al. found that the tumor microenvironment promotes TLS formation and is correlated with enhanced angiogenic potential (70). Liang et al. demonstrated that the maturity of TLS-particularly follicular TLS-serves as a potential mediator of anti-tumor immunity in laryngeal cancer (71). In addition, TLS serves not only as an indirect biomarker for effective antitumor responses in laryngeal cancer, but also as a reliable predictor of recurrence risk (72, 73). In summary, the three-TLS functions as an “anti-tumor immune base” in laryngeal cancer. Diagnostically, it serves as a novel indicator for evaluating the tumor immune microenvironment. Therapeutically, it predicts the efficacy of immunotherapy and inspires new treatment strategies; prognostically, it acts as a positive marker for assessing patient survival outcomes. Future in-depth research and clinical application of TLS hold great promise for providing more robust evidence to support personalized, precision immunotherapy in laryngeal cancer.

### 3.4 Characteristics and significance of TLS in other HNC

In addition to the above three types, common head and neck tumors include thyroid cancer and pharyngeal cancer. TLS has been clearly identified in thyroid cancer, where its maturity may correlate with immune regulatory functions. Particularly in anaplastic carcinoma, it may be associated with immunotherapy sensitivity (74, 75). However, research on TLS in other head and neck tumors, such as hypopharyngeal carcinoma, remains in its infancy. Although evidence supporting TLS as a biomarker for clinical decision-making in hypopharyngeal carcinoma and other cancers is currently insufficient, its central role in tumor immunity positions it as a highly promising research direction in this field.

## 4 TLS detection and evaluation methods

**Figure 2:**
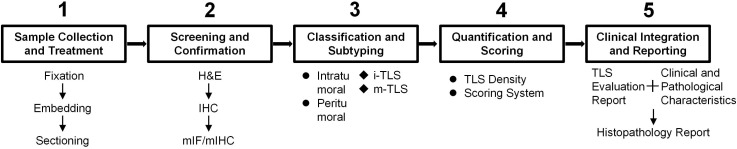
A diagram illustrating the recommended workflow for TLS assessment in clinical. H&E, hematoxylin and eosin; IHC, immunohistochemistry; mIF/mIHC, multiplex immunofluorescence/multiplex immunohistochemistry; TLS, tertiary lymphoid structure; i-TLS, immature TLS; m-TLS, mature TLS.

Accurate and reliable detection and quantification of TLS are prerequisites for advancing it from research to clinical application. Current methods primarily rely on tissue samples and are evolving toward more refined and quantitative approaches (TLS evaluation process as shown in **Figure 2**). In clinical practice, a combination of approaches is typically used to assess TLS according tumor type, clinical stage, treatment goals, and local medical resources. We have provided a detailed summary of the advantages and disadvantages of our TLS detection method (**Table 2**).

**Table 2:** Comparison of TLS assessment methods.

Method	Advantage	Disadvantage
Clinical palpation	Simple, rapid, non-invasive, and low-cost	Low accuracy and high dependence on experience
Ultrasound	Radiation-free, dynamic observation, and capable of evaluating TLS morphology	Highly operator-dependent and poor deep imaging
CT	High resolution, clear display of TLS size.	Exposure to radiation and limited sensitivity to small metastatic lesions
MRI	Radiation-free and capable of assessing lymph node invasion	Long inspection time and high costs
PET-CT	High detection rate and significant value for metastasis assessment	High cost, high radiation dose, and potential for false positives and false negatives
Fine-needle aspiration cytology	Minimally invasive, rapid, with fewer complications, and capable of determining the cytological nature of lymph nodes	The small sample size may lead to false-negative results, complicating lymphoma typing and making it impossible to assess lymph node architecture
Core-needle biopsy	High accuracy	It remains an invasive procedure with risks of bleeding and infection, and poses higher risks for Level III lymph nodes located deep or near blood vessels
Sentinel lymph node biopsy	It enables precise evaluation of lymph node drainage status, avoids unnecessary extensive dissection, and significantly reduces surgical complications	The procedure is technically complex and requires multidisciplinary collaboration, with a potential false-negative rate, and offers limited clinical value in evaluating lymph nodes with confirmed metastasis
Lymph node dissection	Provide the most accurate pathological staging (including lymph node count, metastatic lesion size, extracapsular invasion), with both diagnostic and therapeutic significance	Significant trauma may lead to permanent complications (such as lymphedema, nerve injury, and shoulder dysfunction), and is only indicated for patients with confirmed or highly suspected metastasis
Multigene testing	High-throughput, high-sensitivity	Dependent on nucleic acid quality with missing spatial information
Molecular subtyping	Patient prognosis can be assessed	Heterogeneity and inconsistent standardization
Multiple Immunofluorescence/Immunohistochemistry	*In situ* spatial information, enabling analysis of cellular phenotypes	The number of markers is limited, semi-quantitative
Spatial Transcriptomics	Obtain spatial maps and analyze microenvironments	Technically complex and expensive, with missing protein information
Imaging Mass Cytometry	Deep phenotype analysis	Exorbitant costs, technical complexity, and sample destruction
AI-based detection	High objectivity and reproducibility, high efficiency and throughput, high precision and in-depth analysis	Highly dependent on training datasets, unable to account for biological complexity

### 4.1 Classic methods based on histopathology

Histopathological methods involve direct observation and analysis of tissue sections, including hematoxylin and eosin (H&E) staining, which allows preliminary identification of TLS through morphological recognition of lymphocyte aggregates. This approach is low-cost and straightforward to perform, but it cannot distinguish cell subtypes and relies heavily on the pathologist’s experience, which introduces subjective bias (5). Immunohistochemistry (IHC) and immunofluorescence (IF) utilize specific antibodies to label cells such as CD20^+^ B cells, CD3^+^ T cells, PDPN^+^ fibroblasts, confirming the cellular composition and structure of TLS. This represents the most accurate method for TLS identification currently available (76, 77). Multiplex immunohistochemistry/immunofluorescence (mIHC/mIF) enables simultaneous labeling of multiple proteins on a single section, providing deeper insights into the spatial relationships and interactions among cells within TLS. It serves as a crucial tool for studying the spatial biology of TLS (5, 77, 78).

### 4.2 Gene expression profile-based detection method

These methods indirectly or directly assess TLS by analyzing gene expression, primarily including real-time quantitative PCR (qPCR) indirectly indicates TLS presence by detecting gene expression levels of TLS-associated chemokines (such as CXCL13, CCL19, CCL21) (5). Another approach is RNA sequencing (RNA-seq), which performs transcriptome analysis on tissue samples to calculate expression scores for TLS-associated genes (such as the “12-chemokine score” or specific multi-gene signatures) (79). This enables relative quantification and correlates with prognosis. Compared to histological methods, genomic analysis may offer greater objectivity and is well-suited for efficient evaluation (42).

### 4.3 Emerging high-dimensional spatial technology

To overcome the limitations of traditional methods, technologies such as spatial omics have emerged, enabling the *in situ* analysis of TLS’s cellular phenotypes, gene expression, and spatial location information at unprecedented resolution and throughput. For example, spatial transcriptomics, which measures gene expression profiles in localized regions while preserving tissue spatial positioning, enables detailed characterization of immune cell phenotypes and active signaling pathways both within and outside the TLS (42, 79). 3D imaging, through techniques such as optical sectioning or micro-CT, enables the reconstruction of the three-dimensional structure of TLS, yielding richer information including volume and cell-cell contacts (78). Imaging Mass Cytometry combined with highly multiplexed tissue imaging enables simultaneous detection of over 40 protein biomarkers at the single-cell level, significantly enhancing the ability to resolve the complex cellular composition of TLS (79).

Despite remarkable advances in relevant technologies, the detection of TLS still faces numerous challenges. For instance, significant discrepancies exist across different studies in terms of the marker panels, scoring systems, and technical platforms adopted for the definition, classification, and quantification of TLS, which hinders valid cross-study comparisons of research outcomes. Therefore, the establishment of a fully validated standardized TLS assessment protocol constitutes a critical prerequisite for facilitating its translation into a clinical companion diagnostic tool. Furthermore, all current detection modalities for TLS rely on tumor tissue specimens, which necessitates sample acquisition via invasive biopsy or surgical procedures. For patients who are unable to obtain sufficient tumor tissue due to various reasons or require long-term dynamic monitoring of TLS alterations, such detection approaches present notable limitations. Therefore, the development of imaging techniques enabling non-invasive, *in vivo* detection and quantification of TLS have emerged as a key research direction in this field moving forward.

### 4.4 Evolution of TLS detection and evaluation paradigm driven by artificial intelligence

With the rapid advancement of artificial intelligence (AI) technology, AI-based methods are gaining increasing attention in the field of TLS automatic identification and quantitative analysis. Machine learning and deep learning have become core technological supports driving the standardization and automation of TLS evaluation processes. The development of AI offers several key advantages for TLS detection and assessment: (1) Traditional TLS identification relies on pathologists conducting manual observation and counting of H&E or IHC sections, which is not only time-consuming and labor-intensive but also prone to subjective bias. In contrast, AI can achieve automated detection, segmentation, and counting of TLS based on digital pathological images, significantly improving analytical efficiency and result reproducibility, thereby realizing the goal of automated identification and quantification (5, 79, 80); (2) AI technology can efficiently process conventional H&E images while integrating multidimensional data such as mIHC/mIF, spatial transcriptomics, and radiomics (42). Through multimodal data fusion and deep feature extraction, it provides robust technical tools for in-depth analysis of TLS functional status and its correlation with clinical outcomes (5); (3) AI facilitates the establishment of objective and unified TLS evaluation standards, effectively reducing human variability and advancing the standardization of TLS assessment (79, 81). Additionally, combining AI-extracted TLS features with clinical data enables the construction of precise predictive and prognostic models (29); (4) AI can also assist in developing novel detection methods, such as indirectly assessing TLS status through analysis of blood or other body fluid components, thereby providing more diverse approaches and tools for clinical testing (5, 81).

However, AI detection of TLS primarily relies on conventional staining images, aiming to overcome the subjectivity and complexity inherent in traditional image recognition while addressing the limitations of conventional experimental methods in large-scale data analysis. In summary, AI has brought revolutionary potential to TLS detection, enabling objective, efficient, and in-depth analysis, serving as a key technology for advancing TLS from research to clinical practice. However, its development still faces multiple challenges including data availability, algorithmic limitations, and clinical validation (78). Future efforts require interdisciplinary collaboration to build high-quality datasets, develop practical models, and validate their value through clinical studies, ultimately achieving AI-assisted precision immunopathological analysis.

## 5 Strategies for targeted TLS therapy in HNC

**Figure 3:**
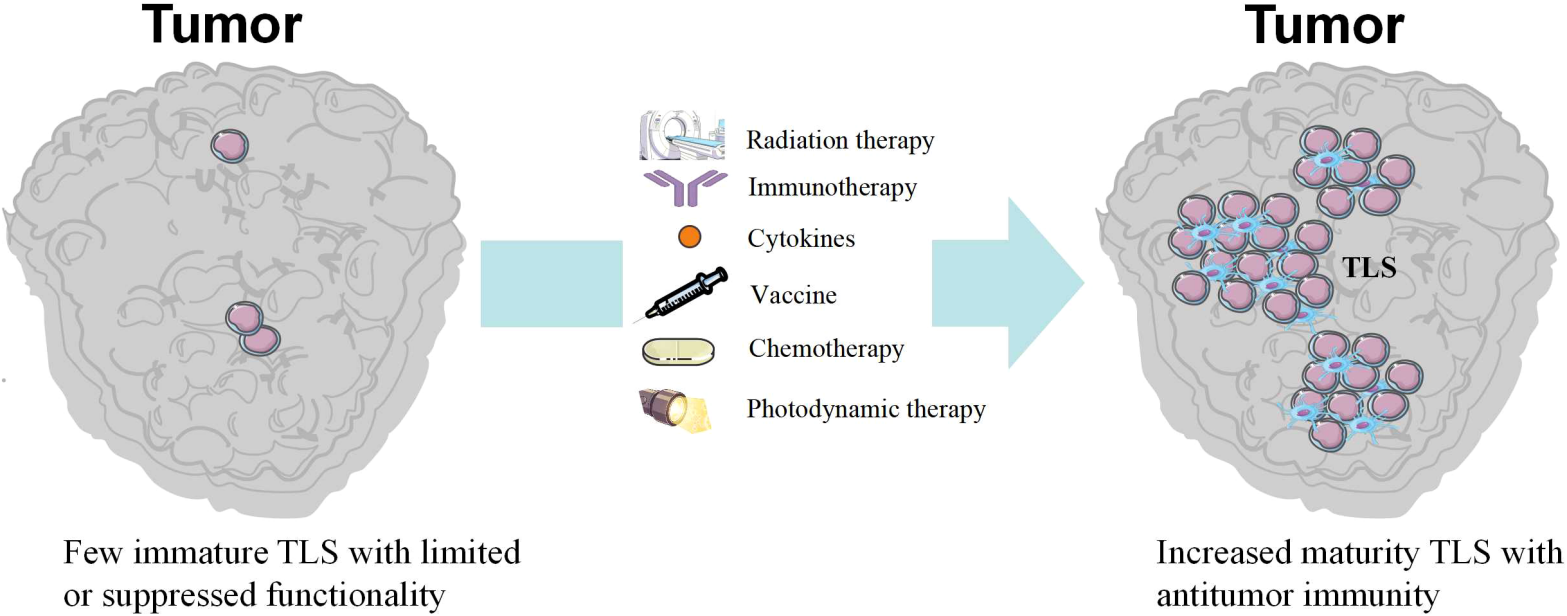
TLS induction strategies. Radiation therapy, immunotherapy, cytokines, vaccine, chemotherapy and photodynamic therapy can induce the formation and maturation of TLS.

Targeting TLS has emerged as a novel strategy in HNC immunotherapy, as its presence correlates with improved prognosis and immunotherapy response. Consequently, inducing TLS formation has become a key therapeutic approach targeting TLS (**Figure 3**). Zhu et al. reported generating TLS *de novo* in HNC by activating lymphotoxin β receptor (LTβR), which promotes lymphoid cell aggregation and HEV formation. Preclinical studies demonstrated that this strategy enhances immune cell infiltration and improves treatment efficacy (4). In addition, local administration or expression of chemokines via vectors (such as CXCL13, CCL19, CCL21) can recruit B cells, T cells, and dendritic cells to promote TLS assembly. Alternatively, cytokines like IL-7 and IL-2 can stimulate lymphoid cell proliferation and aggregation, thereby supporting TLS development (43, 82). In summary, targeting TLS offers a novel approach for immunotherapy in head and neck cancer. By inducing or leveraging these structures to enhance antitumor immune responses, it holds promise for improving response rates to existing immunotherapies and enhancing patient outcomes. We summarized the strategies for combining TLS with immunotherapy (**Table 3**).

**Table 3:** Strategies combining TLS with immunotherapy.

Combination therapy strategies	Clinical logic of combination therapy
Immune checkpoint inhibitors+TLS	PD-1/PD-L1 inhibitors relieve immunosuppression, drive the formation and maturation of tumor-associated lymphocytes (TLS), and amplify the antitumor immune response
Vaccine+TLS	Vaccines deliver antigens, while TLS provides an immune-activating microenvironment to achieve efficient antigen presentation and cellular activation.
CAR-T+TLS	CAR-T cells kill tumor cells while TLS enhances endogenous immunity, overcoming immune exhaustion following CAR-T therapy.
Oncolytic virus+TLS	Oncolytic viruses lyse tumors, release inflammatory signals, induce TLS reactivation, and reverse the immune desert microenvironment

## 6 Current status and future prospects of TLS application in clinical diagnosis and treatment

In recent years, with the rise of immunotherapy, the value of TLS in clinical diagnosis and treatment of tumors has become increasingly prominent. Firstly, TLS can be utilized for prognostic assessment in cancer patients-numerous studies have demonstrated that the presence of TLS within or peritumors, particularly structurally intact and functionally mature TLS, is significantly associated with longer patient survival (78, 83). Additionally, as a key biomarker for responses to immunotherapies such as ICIs, TLS whether pre-existing or newly formed post-treatment is closely linked to higher response rates and prolonged survival benefits in ICIs therapy (78, 84).

TLS serves not only as a significant biomarker but also as an emerging therapeutic target. For instance, inducing TLS formation through chemokines, chemical agents, or vaccines can transform “cold tumor” into “hot tumor, “ thereby enhancing immune therapy sensitivity (77, 85, 86). Despite promising prospects, the clinical translation of TLS faces challenges: firstly, the standardization of TLS histological scoring and large-scale clinical validation have not been effectively advanced; secondly, TLS exhibits high heterogeneity, necessitating the establishment of a more refined classification system (42, 87).

In summary, TLS has rapidly evolved from a mere pathological phenomenon into a highly valuable biomarker and emerging therapeutic target in the field of tumor immunotherapy. Currently, it is widely utilized for prognosis assessment and efficacy prediction. In the future, standardized detection and therapeutic strategies that actively induce TLS formation are expected to fundamentally improve clinical outcomes in patients with immunotherapy insensitivity, ushering in a new era of personalized immunotherapy.

## 7 Conclusion

In conclusion, TLS have been identified as critical regulators of the tumor immune microenvironment in HNC, with specific mechanisms and clinical implications. Clinical studies have demonstrated that the formation of TLS is associated with improved survival rates in HNC patients. Mechanistically, TLS assembly is driven by chemokines and cytokines, which recruit tissue immune cells to form functional germinal centers (GCs), thereby generating high-affinity antibodies and sustaining a prolonged antitumor immune response. Treatment strategies targeting TLS have demonstrated promising prospects in preclinical models, with combination therapies (TLS induction + ICIs, vaccines, or CAR-T) potentially overcoming the “immune desert” phenotype in advanced head and neck cancers. These advancements position TLS as a core component of precision immunotherapy, offering the potential to revolutionize the treatment of head and neck cancers by enhancing response rates and improving long-term patient outcomes.
